# The Non-Steady State Growth of Pearlite outside the Hultgren Extrapolation

**DOI:** 10.3390/ma9120998

**Published:** 2016-12-14

**Authors:** Maria Martin-Aranda, Rosalia Rementeria, Robert Hackenberg, Esteban Urones-Garrote, Shao Pu Tsai, Jen Ren Yang, Carlos Capdevila

**Affiliations:** 1 Tata Steel Research and Development, Swinden Technology Centre, Rotherham S60 3AR, UK; Maria.Aranda@tatasteel.com; 2 Materalia Research Group, National Center for Metallurgical Research (CENIM-CSIC), 28040 Madrid, Spain; rosalia.rementeria@cenim.csic.es; 3 Materials Science and Technology Division, Los Alamos National Laboratory (LANL), Los Alamos, NM 87545, USA; roberth@lanl.gov; 4 Centro Nacional de Microscopía Electrónica (CNME), Facultad de Ciencias Químicas, Universidad Complutense de Madrid, Avda. Complutense s/n, 28040 Madrid, Spain; esteban.urones@pdi.ucm.es; 5 Department of Materials Science and Engineering, National Taiwan University, Taipei 10617, Taiwan; sptsai@ntu.edu.tw (S.P.T.); jryang@ntu.edu.tw (J.R.Y.)

**Keywords:** pearlite, partitioning, non-steady state, thermodynamics, divergence

## Abstract

The goal of this paper is to analyse the effect of adding Al on the non-steady pearlite growth occurring in a Fe–C–Mn system. The results are discussed in terms of the partitioning of elements across the austenite/ferrite and austenite/cementite interfaces, and the modification of the pearlite driving force related to the change in carbon activity in austenite.

## 1. Introduction

The decomposition of austenite is one of the most technologically important phase transformations, since the kinetics of these reactions determine to a large degree the properties of the steel. From a fundamental point of view, seminal works of Hillert [1], Mehl [2], and Hultgren [3] allow us to understand the kinetics of austenite-to-pearlite transformation in Fe–C alloys. On the other hand, a number of attempts have been made to study the effect of ternary additions on the kinetics of pearlite transformation [4,5,6,7,8]. This addition alters the formation of pearlite by affecting the nucleation and growth rates [2]. All alloying elements in steels, with the exception of Co, retard pearlite transformation kinetics [8].

Nevertheless, the role of an austenite stabiliser such as Mn deserves special attention. The addition of Mn induces at specific three-phase austenite (γ) + ferrite (α) + cementite (θ) regions, where austenite and pearlite coexist in equilibrium [9]. Hutchinson et al. [10] studied the isothermal growth of partitioned pearlite in a series of high-purity Fe–C–Mn alloys. They reported two types of pearlite morphologies and determined the Mn distribution across the interface and in the product phases by the use of analytical transmission electron microscopy (ATEM). The authors concluded that for the alloy transformed within the (α + θ) two-phase field, the Mn contents inherited by the growing α and θ were remarkably constant for much of the reaction. This is consistent with the fact that the driving force for the reaction is constant; hence, pearlite grows under steady-state conditions [1,10]. Thus, pearlite reaction proceeds with constant interlamellar spacing and a growth rate, consuming 100% of the parent austenite. In the case of the alloy transformed within the (γ + α + θ) three-phase field, the Mn contents inherited by the growing α and θ both increased with time, which is explained on the basis of the driving force continuously decreasing while the reaction takes place, thereby decreasing the growth rate and increasing the interlamellar spacing, also leading to a pearlite volume fraction lower than 100% [1,10,11]. Cahn and Hagel used the name of divergent pearlite to describe this pearlitic morphology [11]; later, Hillert provided a thermodynamic description of this experimental observation [1].

Additionally, the partitioning of the substitutional solutes between ferrite and cementite has a strong effect on the growth mechanism. It is now generally agreed that, during pearlite growth, the partitioning of alloying elements between ferrite and cementite occurs at low supersaturations, and the growth is controlled by boundary diffusion. By contrast, at high supersaturations, pearlite growth occurs without any partitioning of the alloying element, and the growth is controlled by carbon volume diffusion. These two growth modes are known as partition local equilibrium (PLE) and as non-partition local equilibrium (NPLE), respectively [12,13,14]. Hultgren coined the term “para-pearlite” for any eutectoid structure in which the partitioning of substitutional elements between product phases cannot be expected, and “ortho-pearlite” for structures in which the alloying element partitioning occurs according to phase equilibria [3]. Austenite stabiliser elements, e.g., Ni, have low solubility in cementite and will encourage the non-partitioning reaction. By contrast, those elements which are strong ferrite formers, e.g., Cr, Mo, are soluble in cementite, and hence are likely to exhibit a partitioning type reaction at a higher transformation temperature [15].

Our goal in this paper is to analyze the role that the addition of Al has on divergent pearlite in a Fe–C–Mn system. This aim will be accomplished following a two-fold strategy—firstly, by analyzing the growth rate, the interlamellar spacing variation with the reaction time, and the evolution of the α and θ composition measured at the interface with the austenite during the reaction. These three aspects can help us to study and quantify the divergence phenomenon. Secondly, the change in the driving force, which is related to the change in the carbon activity in austenite, will be tracked.

## 2. Materials and Experimental Procedure

Two high-purity alloys were prepared by vacuum induction melting and supplied as 20 kg ingots by Acerinox Company. Ingots were homogenised at 1050 °C for 24 h and further hot-forged and annealed to ensure uniformity in the composition of the steel. The chemical compositions for the Fe–C–Mn and the Fe–C–Mn–Al (henceforth Mn-steel and Al-steel) are presented in Table 1. Fe composition is to balance (bal.).

The thermodynamic calculations in both cases were performed using Thermo-Calc and TCFE 7 databases. Isothermal heat treatments were performed in cylindrical samples 6 mm in diameter and 20 mm in length using a tube furnace. Samples were subsequently water quenched to room temperature. A 2% picral solution was used to reveal the microstructure for observation via optical microscopy (OM) and scanning electron microscopy (SEM). SEM characterization was performed with a Hitachi S 4800 J field emission gun scanning electron microscope (FEG-SEM) operating at 10 kV.

The Mn and Al concentration across the γ/α and γ/θ interfaces was performed by means of Analytical Transmission Electron Microscopy (ATEM). A JEOL 3000F TEM equipped with an Oxford INCA energy dispersive X-ray spectrometer (EDS) system was used. Standard 3 mm diameter TEM disks were mechanically thinned and then electropolished using a 10 vol % perchloric/acetic acid solution at room temperature and a potential of 40 V.

## 3. Results and Discussion

**Figure 1:**
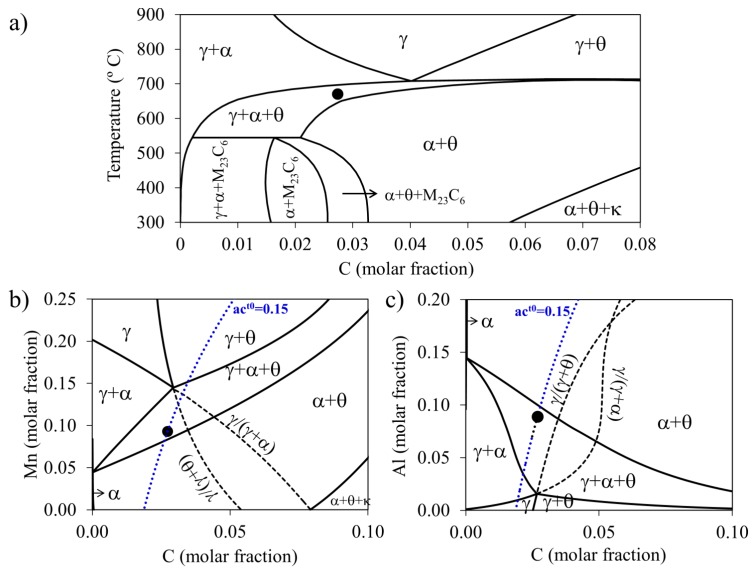
(**a**) Isopleth section of the Fe–C–Mn–Al system at 0.0930 Mn–0.0886 Al (molar fraction), showing that at 670 °C the alloy composition (represented by the solid circle) is located within the three-phase (γ + α + θ) field region. Isothermal section at 670 °C for the studied steel at (**b**) 0.0886 Al (molar fraction) and (**c**) 0.0930 Mn (molar fraction) fixed. The blue dotted line is the carbon isoactivity line at the early stages of the transformation (ac^t0^) and the black dashed lines are the extrapolated γ/(γ + α) and γ/(γ + θ) phase boundaries (κ symbolises Kappa-carbide and it does not participate in reactions in this study).

**Figure 2:**
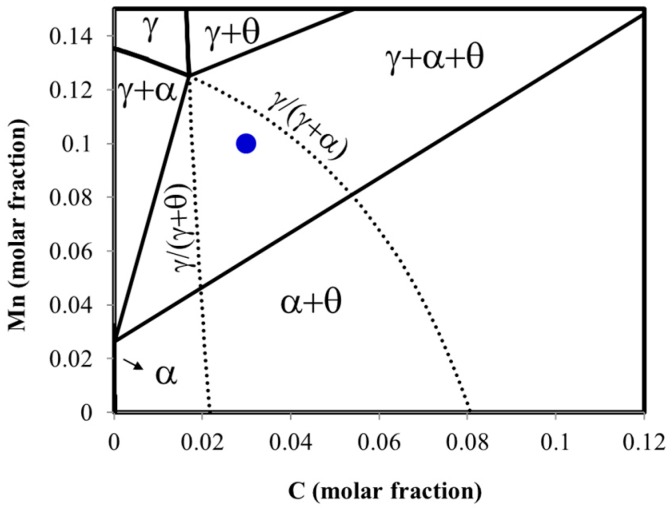
Isothermal section at 600 °C for the studied Mn-steel at 0.0966 Mn fixed. The black dashed lines are the extrapolated γ/(γ + α) and γ/(γ + θ) phase boundaries.

On the basis of the isopleth section for Al-steel (see Figure 1), the isothermal heat treatments were performed at 670 °C at different times with the aim of obtaining interrupted stages of divergent pearlite formation. Additionally, the isothermal sections for the selected temperature are shown in Figure 1b,c, where it is observed that the alloy composition lies within the three-phase field. In the case of Mn-steel, the selected temperature was 600 °C (see Figure 2) following an equivalent reasoning as the above for Al-steel as it was reported elsewhere [16].

**Figure 3:**
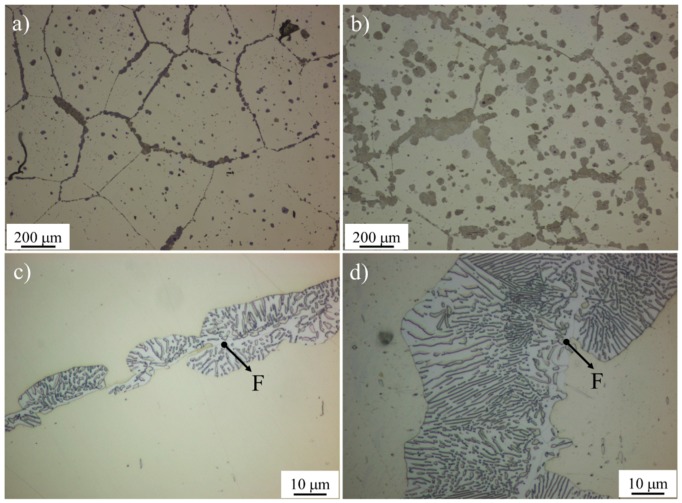
Optical micrographs showing the microstructural evolution during pearlite reaction for the Al-steel after heat treatment at 670 °C for (**a**,**c**) 3 days and (**b**,**d**) 13 days. In (c,d), it is evident that the volume fraction of proeutectoid ferrite (F) is negligible; moreover, over the reaction time, divergent pearlite can be highlighted. In (a,b), the dark areas correspond to pearlite, and the matrix is austenite. The lamellar structure in (c,d) corresponds to pearlite. Proeutectoid ferrite (F) is highlighted.

An example of the microstructure obtained after isothermal heat treatment at 670 °C for 3 and 13 days in Al-steel are presented in Figure 3a,b. After 13 days, the achieved volume fraction of pearlite was around 30%. It is worth mentioning that, at 670 °C, the Al-steel was located outside the Hultgren extrapolation (see Figure 1b,c), which is the area enclosed by the extrapolation of the γ/(γ + α) and γ/(γ + θ) phase boundaries [3,17]. For this situation, it was assumed that proeutectoid ferrite must be the first product to form, and pearlite will be formed when the composition of the remaining austenite reaches the curve Acm in the phase diagram, i.e., the pair carbon content and temperature values at which primary cementite stars to form. Some authors reported recently that almost fully pearlitic microstructures can be obtained, even for austenite compositions lying outside the Hultgren area [18,19]. It was concluded that pearlite can form over a much wider span of average austenite carbon concentrations than those defined by the Hultgren extrapolation, provided that some amount of the more favourable proeutectoid phase is present. Once initiated, pearlite can grow into austenite having any composition inside the (α + θ) two-phase field. Pearlite formation outside of the Hultgren extrapolation is most readily observed in coarse-grained austenite, where the amount of proeutectoid constituent is minimised relative to that of pearlite, according to the hypothesis that austenite grain size affects the final volume fractions of proeutectoid phase and pearlite. Following the same reasoning, the prior austenite grain size used in this work was very large (450 ± 50 μm), with the intention of diminishing the nucleation sites for proeutectoid ferrite and hence promoting the pearlite formation. From Figure 3c,d, it is evident that the volume fraction of proeutectoid ferrite (F) is negligible (<1%) and that lamellar pearlite is obtained as the major constituent. In addition, lamellar divergence can be perceived across the pearlitic nodules, being more pronounced with longer isothermal decomposition times.

**Figure 4:**
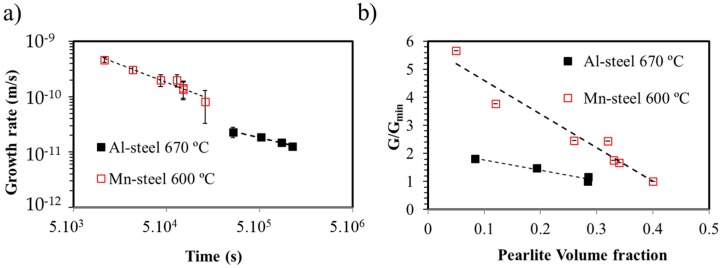
(**a**) Evolution of the growth rate of pearlite with time decomposition at 670 °C for the Fe–C–Mn–Al system and at 600 °C for the Fe–C–Mn system; (**b**) growth rate vs. pearlite volume fraction for the Mn- and Al-steel.

In order to confirm that, for the experimental conditions selected, pearlite is growing under a non-steady state regime, the growth rate of pearlite must be evaluated. Growth rate measurements were carried out using the method proposed by Cahn and Hagel [11] and used by other authors to characterise divergent pearlite [10]. The results for the growth kinetics of the pearlite formation in Al-steel at 670 °C (as well as for the Mn-steel at 600 °C) are presented in Figure 4a. It is shown that the growth rate for both steels decreases as the transformation proceeds, indicating that the growth is under non-steady state conditions. Figure 4 shows the evolution of the pearlite growth rates for the Mn-steel and the Al-steel. For the sake of easier comparison, Figure 4b shows normalised pearlite growth rates by the minimum growth rate measured (G/G_min_). The prevailing tendency of the experimental data shown in Figure 4b is the straight line. The slope indicates that the non-steady-state growth is the governing pearlite growth mechanism in these two systems. It can be concluded, therefore, that the non-steady-state growth is less pronounced in Al-steel as compared with the Mn-alloy. With a very similar volume fraction of pearlite, the pearlite kinetics are faster in the Fe–C–Mn system than in the Fe–C–Mn–Al system.

**Figure 5:**
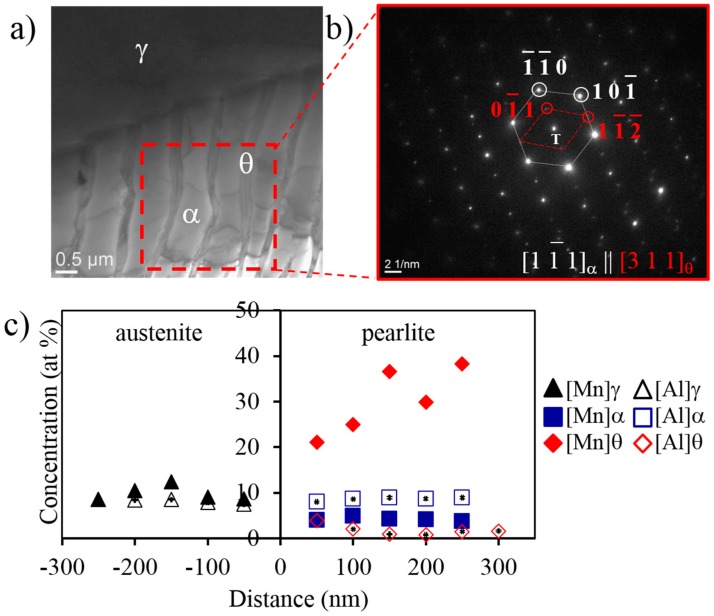
(**a**) TEM image showing the γ/α and γ/θ interfaces in pearlite obtained at 670 °C after 3 days. White dashed lines indicate where the measurements were carried out; (**b**) the corresponding selected area diffraction pattern for the pearlite colony presented in (a)—(white) the diffraction spots from ferrite and (red) from cementite; (**c**) ATEM measurements of Mn and Al across the γ/α and γ/θ interfaces.

**Figure 6:**
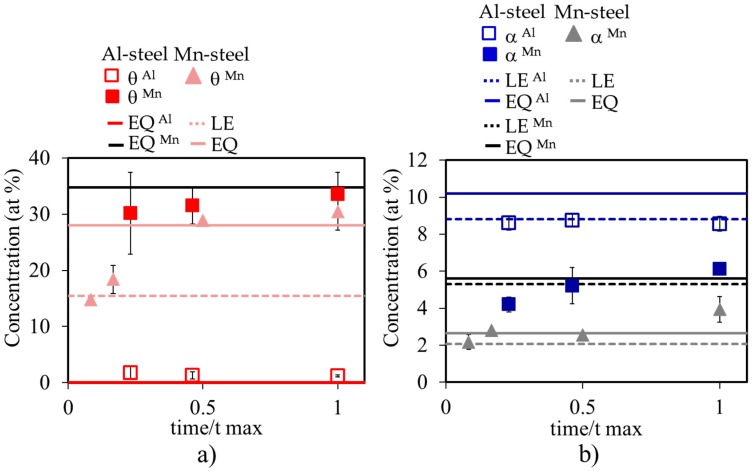
(**a**) Mn and Al concentrations (solid and hollow squares, respectively) for the Al-steel and Mn concentration (triangles) for the Mn-steel in cementite and (**b**) in ferrite during different holding times at 670 °C. Theoretical values predicted by Thermo-Calc are also represented: solid lines are the equilibrium values for Al and Mn in cementite and in ferrite. LE values are given by the dotted lines.

As mentioned above, the divergence might be explained by considering the evolution of the local austenite composition at the γ/α and γ/θ interfaces during the reaction. Therefore, the study of the partitioning of the alloying elements between austenite and the growing pearlite must be addressed. An example for the analysis procedure of an interface is presented in Figure 5. Figure 5a shows a TEM image for γ/α and γ/θ interfaces obtained after decomposition at 670 °C for three days. The corresponding selected area diffraction pattern is shown in Figure 5b, where α and θ are indexed. (As an additional comment, α and θ hold the typical crystallographic orientation relationship (Isaichev’s relationship) reported in the literature [20,21,22]). The Al and Mn concentrations in each phase (γ, α, and θ) are collected in Figure 5c. This graph presents the partitioning trends of Mn (solid points) and Al (hollow points) across the interface for samples treated at 670 °C for 3 days. The ATEM results summarizing the Mn and Al redistribution profiles across the relevant interfaces at different holding times at 670 °C are collected in Figure 6 for both Al- and Mn-steels. The Mn and Al values in both phases (α and θ) reported in Figure 6a and in Figure 6b are the average of 2–3 interfaces, where 5–8 individual measurements were done along the mid rib of a lamella, and the errors bars correspond to the standard deviation of these measurements. The final equilibrium values obtained with the Thermo-Calc software for both alloying elements in the pearlitic phases are plotted together with the experimental values. In addition, the LE values for the ferrite phase were calculated according to [23,24]. However, it was not possible to calculate the theoretical LE values for cementite, since the alloy is not supersaturated with respect to this phase, i.e., it lies outside of the γ/(γ + θ) phase boundary.

Based on the experimental ATEM measurements, it is concluded here that Mn increases in Al-steel during transformation in both α and θ, reaching the full equilibrium value after 13 days. This observed partitioning trend is in good agreement with the results reported by other authors [10,16]. On the other hand, in the case of Al-steel, the Al content in cementite is negligible as it could be expected due to its low solubility [25,26]. The concentration of Al in ferrite remains approximately constant and close to the LE values.

The results shown in Figure 6 reveal a substantial difference in the partitioning of Mn and Al during pearlite growth in both Al- and Mn-steels. Meanwhile, equilibrium compositions in ferrite and cementite are reached for the case of Mn-steel; for the case of Al-steel, the Al equilibrium composition in ferrite is not reached after 13 days. Therefore, the analysis of the evolution of local compositions at the γ/α and γ/θ interfaces during growth in Al-steel is not conclusive for determining the divergence in Al-steel according to the expectation of the LE hypothesis [1]. An alternative method is required.

It is well accepted that the reaction of divergent pearlite is observed when the composition of the parent austenite is located within the (γ + α + θ) three-phase region. The fact that the equilibrium composition of the austenite differs from the bulk composition leads to a continuous change on the austenite carbon composition that causes a reduction in the driving force for this transformation. As a result, the interlamellar spacing increases, the growth rate decreases, and the partitioning extent increases with reaction time until austenite reaches equilibrium [1,10,11,16].

At the beginning of the transformation the LE conditions across the γ/α and γ/θ can be described by a single carbon isoactivity line (ac^t0^) that passes through the bulk composition. Then, the composition for ferrite and cementite can be read from the phase diagram selecting the adequate tie-lines. The growing pearlite has a higher C content than the alloy; hence, it draws C from the parent austenite leaving a C-depleted zone. This leads to a reduction in the activity of C in the vicinity of the interface. As the transformation progresses, the LE conditions prevailing at the interface change continuously, and can be described by C isoactivity lines corresponding to lower C activities. The LE values of the growing ferrite and cementite will change in accordance with the variation in the operative carbon isoactivity line and their corresponding tie-lines as the reaction proceeds. When austenite reaches the equilibrium composition with respect to carbon (defined by the corner the stable γ phase field), the LE reaction mode will cease and the composition for the last-formed pearlite would be described by the final carbon isoactivity line (ac^tF^) and the associated tie-lines. Therefore, there is a close relationship between the change in the carbon activity and the observation of the divergence phenomenon. The evaluation between the initial and the final state through the carbon isoactivity value could be an effective way to study and justify divergent pearlite formation. In some way, this change in carbon activity is related to the available driving force for the reaction.

It is well-known that the chemical potential of carbon in austenite at the alloy composition ($\mu_{C}^{t0}$) and at the end of transformation ($\mu_{C}^{tF}$) could be described as a function of the carbon activity ($a_{C}$) as follows:

(1) $$\mu_{C}^{t0}=\mu^{0}+RTa_{C}^{t0}$$

(2) $$\mu_{C}^{tF}=\mu^{0}+RTa_{C}^{tF}$$

where $\mu^{0}$ represents the chemical potential of carbon in austenite in a standard state. At the beginning and end of the transformation, the standard state for carbon is considered to be the same. The change in chemical potential of carbon in austenite during the pearlite reaction ($\Delta \mu =\mu_{C}^{tF}-\mu_{C}^{t0}$), which is directly related to the available driving force for the reaction, could be expressed as

(3) Δμ=RTln(aCtFaCt0).

For this purpose, the activity of carbon for Al- and Mn-steel, among other alloys reported in literature where divergent pearlite was identified, has been calculated and is listed here in Table 2.

From the results presented in Table 2, it can be deduced that the change in carbon isoactivity presented in this work is half an order of magnitude lower than in other works where divergent pearlite was observed. It might be concluded from the results listed in Table 2 that the driving force for divergent pearlite is significantly lowered with the addition of Al to a Mn-steel, which is clearly found in a non-steady state pearlite growth regime. Based on the experimental data and observations of this work, the divergence phenomenon in a Fe–C–Mn–Al steel treated at 670 °C is subtle. In spite of the fact that the alloy composition lies within the three-phase region, the small change in carbon activity (driving force), the small differences in Mn and Al concentrations in the pearlitic phases, and the slight differences in the growth rate make it difficult to clearly observe divergent pearlite.

## 4. Conclusions

In summary, the results presented in this work and the corresponding analysis and discussion, allow us to obtain the following main conclusions:

1. Pearlite can nucleate from a much wider span of average austenite carbon concentrations than those defined by the Hultgren extrapolation, provided that some amount of the more favourable proeutectoid phase is present. Once initiated, pearlite can grow into austenite having any composition inside the α + θ two-phase field and the γ + α + θ three-phase field. As has been reported previously by the authors [16,19], the flux balance ahead of the interface allows lamellar pearlite formation outside Hultgren extrapolation.
2. The experimental ATEM measurements allow us to conclude that Mn increases in Al-steel during transformation in both ferrite and cementite, reaching the full equilibrium value after 13 days. On the other hand, in the case of Al-steel, the Al content in cementite is negligible, as was expected due to its low solubility. The concentration of Al in ferrite remains approximately constant and close to the LE values.
3. The change in carbon activity in Al-steel is smaller than that for the Mn-steel in the same (γ + α + θ) three-phase region of the corresponding phase diagram, which explains the lower driving force for the pearlite reaction.
4. All these results allow us to conclude that divergent pearlite can be observed not only when the alloy composition lies within the (γ + α + θ), but also when the change in carbon activity is large enough to make the divergent microstructure noticeable.

## Figures and Tables

**Table 1:** Chemical composition in wt % and at % for the Mn-steel and Al-steel.

Steel	Unit	C	Mn	Al	Fe
Al-steel	wt %	0.63	9.82	4.60	bal.
	at %	2.74	9.30	8.86	bal.
Mn-steel	wt %	0.65	9.72	-	bal.
	at %	2.98	9.66	-	bal.

**Table 2:** Values for the activity of carbon in the austenite at the beginning (ac^t0^) and at the end (ac^tF^) of the divergent pearlite transformation for different system alloys and isothermal temperatures (T), all of which were transformed in the γ + α + θ three-phase field. Δac and a* are the difference between ac^tF^ and ac^t0^, and the ratio between ac^tF^ and ac^t0^ (ac^tF^/ac^t0^), respectively.

Alloy (wt %)	T (°C)	ac^t0^	ac^tF^	Δac	a*	Δμ (J·mol^−1^)	Reference
Fe–0.6C–5.2Mn	650	0.250	0.182	−0.068	0.728	−2439	[11]
Fe–0.6C–5.2Mn	625	0.300	0.156	−0.144	0.520	−4869	[11]
Fe–1C–5Mn	680	0.420	0.214	−0.206	0.509	−5339	[27]
Fe–0.6C–4.7Mn	650	0.260	0.182	−0.078	0.700	−2744	[10]
Fe–0.55C–5.4Mn	625	0.270	0.156	−0.114	0.577	−4080	[10]
Fe–0.49C–6.3Mn	600	0.270	0.132	−0.138	0.489	−5197	[10]
Fe–0.42C–6.8Mn	575	0.265	0.109	−0.156	0.411	−6256	[10]
Fe–0.66C–9.74Mn	600	0.320	0.130	−0.190	0.406	−6543	[16]
Fe–0.6C–9.8Mn–4.6Al	670	0.150	0.130	−0.020	0.867	−1123	CW **

** CW stands for current work.
